# Trimellitic anhydride-conjugated serum albumin activates rat alveolar macrophages *in vitro*

**DOI:** 10.1186/1745-6673-1-13

**Published:** 2006-06-23

**Authors:** Dingena L Valstar, Marcel A Schijf, Erietta Stelekati, Frans P Nijkamp, Nanne Bloksma, Paul AJ Henricks

**Affiliations:** 1 Pharmacology and Pathophysiology, Utrecht Institute for Pharmaceutical Sciences, Faculty of Science, Utrecht University, Utrecht, The Netherlands; 2 Deparment of Biology, Faculty of Science, Utrecht University, Utrecht, The Netherlands

## Abstract

**Background:**

Occupational exposure to airborne low molecular weight chemicals, like trimellitic anhydride (TMA), can result in occupational asthma. Alveolar macrophages (AMs) are among the first cells to encounter these inhaled compounds and were previously shown to influence TMA-induced asthma-like symptoms in the Brown Norway rat. TMA is a hapten that will bind to endogenous proteins upon entrance of the body. Therefore, in the present study we determined if TMA and TMA conjugated to serum albumin induced the production of the macrophage mediators nitric oxide (NO), tumour necrosis factor (TNF), and interleukin 6 (IL-6) *in vitro *using the rat AM cell line NR8383 and primary AMs derived from TMA-sensitized and naïve Brown Norway rats.

**Methods:**

Cells were incubated with different concentrations of TMA, TMA conjugated to bovine serum albumin (BSA), and BSA as a control for 24 h and the culture supernatant was analyzed for mediator content.

**Results:**

TMA alone was not able to induce the production of mediators by NR8383 cells and primary AMs from sensitized and sham-treated rats. TMA-BSA, on the contrary, dose-dependently stimulated the production of NO, TNF, and IL-6 by NR8383 cells and of NO and TNF, but not IL-6, by primary AMs independent of sensitization.

**Conclusion:**

Results suggest that although TMA is a highly reactive compound, conjugation to a suitable protein is necessary to induce mediator production by AMs. Furthermore, the observation that effects of TMA-BSA were independent of sensitization suggests involvement of an immunologically non-specific receptor. In the discussion it is argued that a macrophage scavenger receptor is a likely candidate.

## Background

Trimellitic anhydride (TMA) is a reactive low molecular weight (LMW) chemical used in the manufacture of paints, epoxy curing agents, printing inks and vinyl plasticizers and is known to cause occupational asthma characterized by airflow obstruction, airway inflammation and non-specific bronchial hyperreactivity [1-3]. The development of this allergic disease requires sensitization triggered by dermal or respiratory exposure to TMA followed by its binding to proteins [4]. These so formed TMA-protein conjugates will then be taken up by antigen-presenting cells, transported to the regional lymph node, and presented to TMA-specific T cells resulting in T cell memory and the production of TMA-specific IgE antibodies. These antibodies will then bind to the high-affinity IgE receptor on mast cells [5,6] and upon renewed contact with TMA mediate cross-linking of the IgE-receptors with subsequent release of mediators, that in their turn cause bronchoconstriction and attraction of inflammatory cells [7-9].

Alveolar macrophages (AMs) are among the first cells that encounter inhaled small particles and chemicals in the airways, since these cells are located at the interface between air and lung tissue. AMs are long-lived cells belonging to the family of mononuclear phagocytes. They represent a non-specific cellular host defence mechanism and can do so by binding to and ingestion of micro-organisms and macromolecules via pattern recognition receptors, and the secretion of a broad repertoire of mediators that regulate inflammatory and immune reactions in the lung [10-14]. Previously, it has been shown that depletion of AMs in TMA-sensitized Brown Norway (BN) rats prior to inhalation challenge with TMA, but not TMA-BSA, resulted in ameliorated lung function during the challenge [15,16]. Furthermore, an increased influx of inflammatory cells into the lung lumen was observed 24 h after challenge with TMA as well as TMA-BSA in AM-depleted rats compared to non-depleted control rats [15,16]. Therefore, we investigated the direct effects of TMA and TMA-BSA on the production of nitric oxide (NO), tumour necrosis factor alpha (TNF), and interleukin 6 (IL-6) by AMs using the rat AM cell line NR8383 and AMs derived from TMA-sensitized and naïve BN rats.

## Methods

### Materials

TMA (97% purity) was obtained from Aldrich (Brussels, Belgium) and acetone (HPLC grade) from Merck (Darmstadt, Germany). Highly refined olive oil, 2,4,6-trinitrobenzene sulphonic acid, bovine serum albumin (BSA; cell culture tested), sulphanilamide, and naphthyl-ethylenediamide were purchased from Sigma (St. Louis, MO). Sodium pentobarbitone was obtained from Cevasante Animale B.V. (Maassluis, the Netherlands). K-medium contained Dulbecco's Modified Eagle Medium (DMEM; Cambrex BioScience, Verviers, Belgium) supplemented with 10% fetal calf serum (FCS; Invitrogen BV, Breda, the Netherlands), 10 mM HEPES (Merck), 4 mM L-glutamine, 1 mM sodium pyruvate, 100 U/ml of penicillin, 100 mg/ml of streptomycin, 0.05 mM β-mercapto-ethanol (all from Sigma) and 100 mg/ml gentamycin (Invitrogen). Ham's F12 medium was obtained from Invitrogen, lipopolysaccharide (LPS; *E. coli *O111:B4) from Sigma, and IFN-γ from Genentech Inc. (San Francisco, CA). The TNF and IL-6 ELISA kits were purchased from R&D Systems Inc. (Minneapolis, MN).

### Animals

Female, inbred Brown Norway/CrlBR rats (BN; 7–8 weeks of age) were purchased from Charles River (Maastricht, the Netherlands). The animals were acclimatized at least 5 days before the start of the study. They were kept under conventional laboratory conditions and received food (Tecnilab BMI, Helmond, the Netherlands) and tap water *ad libitum*. All animal procedures were conducted in accordance with the Animal Ethics Committee of Utrecht University (Utrecht, the Netherlands).

### Sensitization procedure

TMA was applied at a concentration of 50% (w/v) in a vehicle solution of 4:1 (v/v) acetone and olive oil. Animals received 150 μl on each flank (approximately 12 cm^2 ^each), which had been shaved with an electrical razor 2–3 days earlier. Seven days after the first sensitization the animals received 75 μl of a 25% TMA solution on the dorsum of both ears. Control animals received vehicle solution. Increased TMA-specific IgE serum levels verified the sensitization status of the TMA-sensitized rats [15,16].

### Preparation of TMA-BSA conjugate

The TMA-BSA conjugate was prepared under aseptic conditions by dissolving 10 mg/ml of BSA in 0.1 M sodium borate buffer (pH 9.4), adding approximately 1.5 mg TMA per ml BSA-solution and stirring at room temperature. After 1 h the same amount of TMA was added and the mixture was stirred for 2 h at room temperature. After centrifugation at 390 × *g *for 5 min, the supernatant was dialyzed successively against phosphate-buffered saline (PBS) and distilled water for 24 h at 4°C. The conjugate was lyophilized and stored at 4°C until use. The degree of substitution of the TMA-BSA was assessed by determination of remaining free amino groups by reaction with 2,4,6-trinitrobenzene sulphonic acid as described previously [17]. The conjugate substitution ratio was approximately 40 mol TMA to 1 mol of BSA.

### Cell culture and stimulation

AMs from TMA-sensitized and naïve rats were obtained by lung lavage at day 20 after treatment. Rats were killed with an overdose of sodium pentobarbitone (0.6 g/kg, i.p.). A cannula was inserted into the trachea, the lungs were lavaged 4 times with 8 ml aliquots of PBS warmed to 37°C and the lavage fluid was immediately thereafter put on ice. The cells were collected by centrifugation for 10 min at 390 × *g *(4°C). After washing 3 time swith PBS, the cells were resuspended in K-medium and incubated at 37°C for 2 h in 100 ml culture flasks (Greiner Bio-One, Alphen a/d Rijn, the Netherlands). After washing away non-adherent cells, the adherent cells (AMs) were scraped off in fresh K-medium supplemented with 1% FCS, adjusted to 1 × 10^6 ^cells/ml, and seeded in a total volume of 100 μl into sterile flat-bottom 96-wells plates (Costar, Cambridge, MA). After 1 h, 25 μl medium, stimulants (2 μg LPS admixed with 10 U IFN-γ per ml, 0.1–3 mg/ml of TMA-BSA, 0.1–3 mg/ml of BSA) in medium, or 10 and 100 μM TMA in 0.01 and 0.1% ethanol were added. All stimulants were filtered through a 0.22 μm syringe-filter (TPP, Trasadingen, Switzerland) before use. After 24 h the supernatants were collected and kept at -20°C until use. Control stimulation of cells with 0.01 and 0.1% ethanol in medium did not affect cells (data not shown).

The AM cell line, NR8383, derived from Sprague Dawley rats, was purchased from the ATCC (Manassas, VA, USA) and maintained in Ham's F12 medium supplemented with 15% FCS, 100 U/ml of penicillin and 100 μg/ml of streptomycin. Cells were subcultured once per week. For that purpose, floating and scraped-off adherent cells were collected by centrifugation, resuspended in fresh medium, and seeded into new culture flasks. For stimulation experiments, both adherent and floating cells were harvested, resuspended in Ham's F12 medium supplemented with 1% FCS, penicillin, and streptomycin, and seeded into flat-bottom 96-wells plates at a density of 1 × 10^6 ^cells/ml. After 1 h, test compounds were added and the cells were further incubated for 24 h as described above. All cells were cultured at 37°C in a humidified atmosphere containing 5% CO_2_. Combinations of several concentrations of LPS and IFN-γ were tested in preliminary experiments to find optimal conditions for maximal production of NO, IL-6 and TNF by the macrophages used. Thereafter, the dose-response effects of TMA and TMA-BSA were measured and compared with the maximal production of these mediators by macrophages used.

### NO measurements

The amount of NO secreted into the culture supernatants was assessed by determination of the concentration of its reaction product, nitrite, using the Griess reaction [18]. In solution, nitric oxide reacts with oxygen to form nitrite and with superoxide anion to form nitrate. Nitrite constitutes for approximately 60% of the total macrophage nitrite and nitrate production [19]. Griess reagent (100 μl of 1% sulphanilamide and 0.1% naphthyl-ethylenediamide in 5% phosphoric acid) was added to 100 μl of sample medium. After incubation at room temperature for 10 min the optical density was measured at 550 nm using a microplate reader (Bio-Rad Laboratories, Hercules, CA). Calibration curves were made with NaNO_2 _dissolved in the culture medium.

### Cytokine assays

Levels of TNF and IL-6 in the culture supernatant of control and stimulated cell cultures were measured using commercial ELISA kits according to the manufacturer instructions. The IL-6 and TNF ELISA kits had detection limits of 10 pg/ml and 15 pg/ml, respectively. A microplate reader was used to measure the optical density at 450 nm.

### Statistical analysis

All data are expressed as mean ± SEM. NO, TNF, and IL-6 levels were statistically analyzed using an unpaired t-test. Differences were considered statistically significant if p < 0.05. Analyses were performed by the usage of Graphpad Prism (version 3.0, San Diego, U.S.A.).

## Results

### Effects of different stimuli on the production of NO, TNF, and IL-6 by NR8383 cells

**Figure 1:**
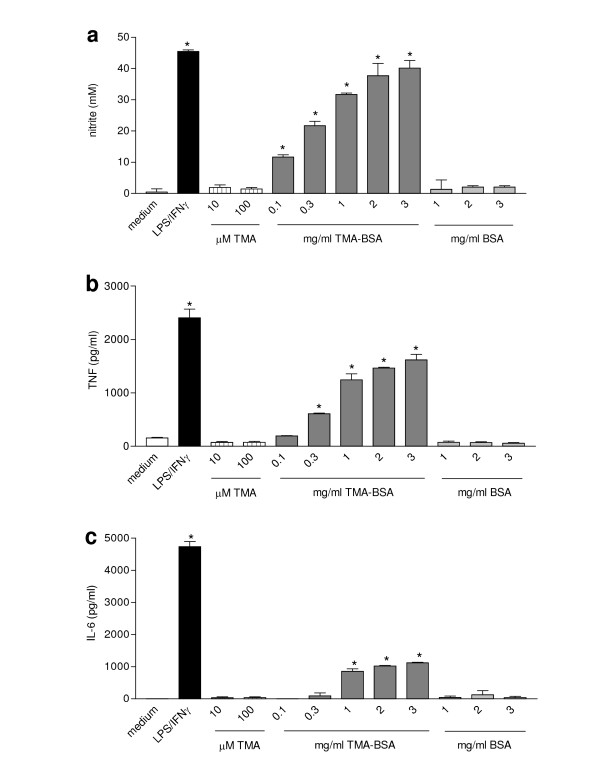
Effect of different stimuli on the production of NO, TNF, and IL-6 by NR8383 cells. Cells were incubated with medium (white bars), LPS/IFN-γ (black bars), TMA (striped bars) TMA-BSA (dark grey bars; 1 mg/ml corresponds with 13.5 μM TMA-BSA) and BSA (light grey bars; 1 mg/ml corresponds with 15.1 μM BSA) for 24 h. The culture supernatants were analyzed for nitrite (a), TNF (b) and IL-6 (c). Results are expressed as mean ± SEM. Significant differences are denoted by *: p < 0.05 compared to medium incubation.

NR8383 cells produced very low levels of NO and TNF and undetectable amounts of IL-6 when cultured for 24 h in medium (Fig. 1). Incubation with LPS/IFN-γ induced the production of TNF and IL-6 already after 6 h (data not shown). After 24 h of incubation, LPS/IFN-γ further increased the production of NO, TNF and IL-6. TMA was not able to stimulate significant production of any of the mediators by NR8383 cells at any time, but TMA-BSA induced TNF and IL-6 production in a concentration-dependent manner after 6 h (data not shown). After 24 h of incubation, TMA-BSA induced the production of NO and further increased the production of TNF and IL-6 production by NR8383 cells in a concentration-dependent fashion. However, the lowest concentrations of TMA-BSA to induce significant production of the separate mediators, and the amounts produced relative to those induced by LPS/IFN-γ diverged. NO was already induced by the lowest concentration of TMA-BSA (0.1 mg/ml) and the NO levels induced by the two highest concentrations were similar to the level induced by LPS/IFN-γ. TNF was induced at TMA-BSA concentrations of 0.3 mg/ml or higher and the maximum TNF level that was induced by 3 mg/ml was approximately 65% of that induced by LPS/IFN-γ. IL-6 was induced at TMA-BSA concentrations of 1 mg/ml or higher and the maximum IL-6 level that was induced by 3 mg/ml was approximately 25% of that induced by LPS/IFN-γ.

NR8383 cells were pre-incubated with 5, 10, and 15% serum derived from either TMA-sensitized or control rats for 1 h to study the effects of passive sensitization with TMA-specific IgE. These treatments did not affect the capacity of LPS/IFN-γ, TMA, TMA-BSA, or BSA to induce the production of NO, TNF, and IL-6 (data not shown)

### Effect of different stimuli on the production of NO, TNF, and IL-6 by AMs from TMA-sensitized and naïve rats

**Figure 2:**
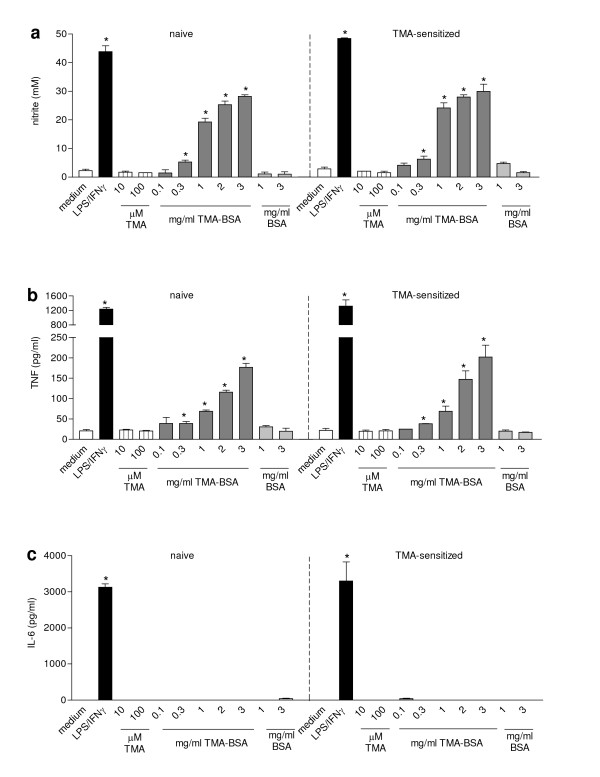
Effect of different stimuli on the production of NO, TNF, and IL-6 by AMs derived from either naïve or TMA-sensitized BN rats. Rats received 150 μl vehicle or 50% TMA in vehicle on each shaved flank on day 0 and 75 μl vehicle or 25% TMA on the dorsum of both ears on day 7. On day 21 the animals were sacrificed and the lungs were lavaged. AMs obtained from the lung lavage fluid were incubated with medium (white bar), LPS/IFN-γ (black bar), TMA (striped bars) TMA-BSA (dark grey bars; 1 mg/ml corresponds with 13.5 μM TMA-BSA) and BSA (light grey bars; 1 mg/ml corresponds with 15.1 μM BSA) for 24 h. The culture supernatants were analyzed for nitrite (a), TNF (b) and IL-6 (c). Results are expressed as mean ± SEM. Significant differences are denoted by *: p < 0.05 compared to medium incubation.

AMs from naïve and TMA-sensitized rats produced very low levels of NO, TNF and undetectable levels of IL-6 when cultured for 24 h in medium. Incubation with LPS/IFN-γ induced equal production of mediators by AMs from both naïve and TMA-sensitized rats (Fig. 2). The LPS/IFN-γ induced NO production by AMs was comparable to that by similarly stimulated NR8383 cells. The LPS/IFN-γ-induced TNF and IL-6 production by AMs, however, was lower than by NR8383 cells. TMA did not induce the production of mediators by AMs after 24 h. TMA-BSA, however, induced a concentration-dependent increase in NO and TNF production by AMs, but did not induce the production of IL-6. Significant levels of both NO and TNF were induced by 0.3 mg/ml of TMA-BSA or more. The maximum NO level that was induced by TMA-BSA was approximately 60% of that induced by LPS/IFN-γ (Fig. 2a) and the maximum TNF level induced by TMA-BSA was approximately 15% of that induced by LPS/IFN-γ (Fig. 2b).

## Discussion

The present study showed that TMA-BSA conjugates, but not free TMA or BSA, were able to induce the production of the mediators NO, TNF, and IL-6 by the cell line NR8383, and, IL-6 excepted, by primary AMs *in vitro*. This stimulation is probably not immunologically specific for two reasons. Firstly, preincubation with serum containing TMA-specific IgE did not affect the capacity of TMA-BSA to stimulate mediator production by NR8383 cells. Secondly, primary AMs from TMA-sensitized and naïve rats reacted similarly to TMA-BSA. The lack of effect of serum from TMA-sensitized BN rats that contained high levels of TMA-specific IgE was not expected, since AMs express the IgE receptors, FcεRI and CD23. Moreover, their expression is increased in the presence of IgE [20] and upon *in vivo *sensitization [21,22]. Apparently, there is no cross-linking of IgE at the surface of AMs by TMA-BSA or cross-linking does not trigger production of the mediators inresponse to TMA-BSA, although similarly prepared conjugates have been reported to trigger degranulation of IgE-primed mast cells [6].

Regarding the nature of the apparent immunological non-specific AM stimulation, structural similarities between TMA-conjugated BSA and maleylated-BSA [23] may point at the involvement of a member of the family of scavenger receptors. These receptors belong to the large family of pattern recognition receptors that exhibit binding specificity for structural patterns typically displayed by cell surface molecules of many micro-organisms [24]. Macrophages are known to express multiple scavenger receptors [25-27] and a variety of ligands, including maleylated-BSA and LPS, have been shown to induce the production of NO, TNF, and IL-6 via scavenger receptors [23,28,29]. Since these mediators were also induced after stimulation of AMs with TMA-BSA, but not free BSA, and because of the structural similarities of TMA-BSA with maleylated-BSA, it is likely that the observed effects were mediated via scavenger receptors.

In the present study, stimulation with LPS/IFN-γ induced the production of higher TNF and IL-6 levels by NR8383 cells than by primary AMs. Since both NR8383 cells and primary cells were AMs, differences in genetic background may explain the variation in mediator production, given the fact that the NR8383 cells were derived from Sprague Dawley rats [30], while the primary AMs were derived from BN rats. Differences in amounts of mediators produced by AMs obtained from Sprague Dawley and from BN rats have been reported [31,32]. Furthermore, differences between primary cells and immortalized cells may be implicated, since Rao et al. [33] demonstrated that stimulation of NR8383 cells with LPS activated three different mitogen-activated protein kinases, while only one of them was activated in primary AMs derived from Sprague Dawley rats.

The observation that TMA-BSA induced equal amounts of NO in NR8383 cells as LPS/IFN-γ and only 40% less in primary AMs indicates that TMA-BSA is a powerful macrophage activating agent. It is probably more potent than LPS as such, since the amount of NO produced by AMs in response to LPS was reported to be only 0–35 % of the response after LPS/IFN-γ incubation of primary AMs derived from Sprague Dawley and BN rats [31]. Despite the potent *in vitro *AM-activating capacity of TMA-BSA and the lack of effect of TMA in this respect, inhalation challenge of TMA-sensitized BN rats with either TMA or TMA-BSA induced similar immediate reduction in minute ventilation [15,16]. However, depletion of AMs prior to challenge of sensitized rats ameliorated the decrease in minute ventilation in case of TMA challenge [15], but not in case of TMA-BSA challenge [16], although both compounds induced an influx of inflammatory cells in the airways of these animals. The substantial differences between TMA and TMA-BSA in their *in vitro *AM-activating capacity are apparently not at play upon inhalation challenge with these compounds. A possible explanation for this controversy might be that inhalation of TMA leads to rapid conjugation to endogenous proteins *in vivo *while formation of such conjugates is not feasible *in vitro *due to the static culture conditions. The observation that TMA challenge of BN rats caused immediate bronchoconstriction [15] is indicative of rapid conjugation, since the immediate bronchoconstriction is likely to be due to mast cell degranulation triggered by IgE receptor cross-linking with a multivalent TMA ligand as formed upon binding of multiple TMA molecules to self-proteins. Since formation of conjugates of TMA with endogenous proteins is considered to be required for sensitization [34] and if such protein-conjugates, like TMA-BSA *in vitro*, induce the production of NO and proinflammatory cytokines *in vivo*, then TMA can be considered as an inducer of danger signals. Thus, TMA-protein-conjugates, like the danger signalling molecules of bacteria, can act as an adjuvant for TMA sensitization. An interesting question in this respect is, whether the most potent inducers of LMW chemical-induced occupational respiratoryallergic disease share this intrinsic adjuvant activity. If so, toxicological hazard identification may benefit from screening for macrophage-activating activity of reactive LMW compounds conjugated with suitable carrier proteins.

## Conclusion

In summary, the results of the present study demonstrate that although TMA is a highly reactive chemical, it needs to be conjugated to suitable protein to exert an effect on mediator production by AMs, as observed for the TMA-BSA conjugate. The effects of TMA-BSA on AMs were not dependent on sensitization, indicating that the interaction of TMA-BSA with AMs is probably mediated via an immunologically non-specific scavenger receptor.

## Competing interests

The author(s) declare that they have no competing interests.

## Authors' contributions

DLV conducted part of the study and was involved in the design of the study, the analysis of the data and the writing of the manuscript. MAS prepared the TMA-BSA and assisted in the study. ES conducted part of the study and was involved in the analysis of the data. FPN helped to obtain the research support and reviewed the manuscript. NB and PAJH obtained the research support and participated in the design of the study, the interpretation of the data and the writing of the manuscript. All authors read and approved the final manuscript.
